# The Complex Interplay of Variables in Extubation Decision-Making Following Pediatric Cardiac Surgery: A Narrative Review

**DOI:** 10.7759/cureus.64216

**Published:** 2024-07-10

**Authors:** Nandha kumar Durai Samy, Karuna Taksande

**Affiliations:** 1 Anesthesiology, Jawaharlal Nehru Medical College, Datta Meghe Institute of Higher Education and Research, Wardha, IND

**Keywords:** complications, extubation outcomes, postoperative mechanical ventilation, early extubation, pediatric, extubation, anesthesia management, endotracheal intubation, congenital heart defects, pediatric cardiac surgery

## Abstract

Pediatric cardiac surgery poses significant challenges in developing countries, where a considerable number of children require intervention for congenital heart disease (CHD). The utilization of endotracheal intubation and anesthesia is pivotal in conducting surgical or angiography procedures on patients with CHD exhibiting diverse anatomical and hemodynamic characteristics. The decision to extubate pediatric patients following cardiac surgery remains a crucial element of postoperative care. This article explores the complexities surrounding extubation decision-making in this population, emphasizing the critical role of surgical, physiological, and postoperative factors. Various preoperative and intraoperative factors influence the timing of extubation. Early extubation is increasingly prevalent, offering benefits like reduced length of stay and minimized drug exposure. Multidisciplinary collaboration and protocol-driven strategies contribute to improved extubation outcomes, emphasizing the need for a comprehensive approach in pediatric cardiac surgery. Future research can focus on the implementation and efficacy of standardized extubation procedures involving collaboration among healthcare experts.

## Introduction and background

Pediatric cardiac surgery poses a significant challenge, as meeting the surgical needs of numerous children with congenital heart disease (CHD) is complicated by the necessity to adopt progressively advanced methods [1]. CHD manifests in four to eight cases per 1,000 live births, with approximately 25% of these instances classified as critical CHDs necessitating intervention within the initial year of life [2]. The utilization of endotracheal intubation and anesthesia is pivotal in conducting surgical or angiography procedures on patients with CHD exhibiting diverse anatomical and hemodynamic characteristics [3]. The primary aim of anesthesia in individuals undergoing surgery for CHD is to uphold hemodynamic stability, minimize mortality and morbidity, optimize resource utilization, and ensure patient safety. Adherence to these criteria is essential when formulating an anesthesia management strategy for CHD cases. Typically, patients undergoing cardiac surgery are monitored in the intensive care unit (ICU) with mechanical ventilation [4,5]. Pediatric cardiac intensive care has undergone significant changes in recent years. There are exciting developments in various aspects of perioperative care that have a direct or indirect influence on postoperative management and results [5]. The rising complexity of diseases, advancements in technology, changing therapeutic approaches, and quality initiatives collectively impose a substantial demand on the healthcare team [6].

In the postoperative phase after pediatric cardiac surgery, the frequent necessity for artificial ventilation underscores a critical consideration. Given the notable complications associated with mechanical ventilation, including the heightened susceptibility to infections, airway and pulmonary injuries, escalated exposure to sedatives and analgesics, and increased utilization of ICU resources, a consensus among clinicians emerges in favor of the proposition that curtailing the duration of postoperative mechanical ventilation (POMV) is indicative of a superior standard of care [7]. In recent years, early extubation following pediatric cardiac surgery has become ever more prevalent in perioperative treatment. Early extubation can potentially reduce the length of hospital stay, minimize complications associated with prolonged ventilator use, decrease patient exposure to drugs and sedation, and provide physiological benefits for specific patient populations. This approach is particularly beneficial for children recovering from cavopulmonary anastomosis or tetralogy of Fallot repair. Numerous studies have demonstrated the success of early extubation techniques across various patient ages and levels of surgical complexity [8].

Current knowledge gaps in the extubation decision-making process following pediatric cardiac surgery highlight several critical areas requiring further investigation. Despite advancements in early extubation protocols and improved postoperative care strategies, the variability in extubation success rates across different institutions underscores the need for standardized guidelines. Moreover, while numerous studies have identified factors influencing extubation outcomes, such as patient-specific variables (e.g., age, weight, and underlying health conditions) and intraoperative considerations (e.g., fluid balance and anesthesia techniques), the precise interplay of these variables remains inadequately understood. Additionally, the long-term neurodevelopmental impacts of early versus delayed extubation and the potential benefits of novel pain management techniques, such as erector spine plane blocks (ESPBs), warrant further exploration. A comprehensive understanding of these complexities is essential to optimize extubation protocols and enhance recovery outcomes for pediatric patients undergoing cardiac surgery.

## Review

Search methodology

To explore "The Complex Interplay of Variables in Extubation Decision-Making Following Pediatric Cardiac Surgery: A Review," a comprehensive search methodology was employed. PubMed, Medline, and Cochrane Library databases were queried for peer-reviewed articles published between 2000 and 2023. Keywords included "pediatric cardiac surgery," "extubation," "variables," "decision-making," "early extubation," and "postoperative care." Inclusion criteria encompassed studies focusing on extubation protocols, outcomes, and related variables in pediatric cardiac surgery patients. Both retrospective and prospective studies, as well as meta-analyses and reviews, were considered. Exclusion criteria were non-English publications and studies with adult subjects. Relevant articles were further screened by titles and abstracts, followed by full-text reviews to ensure the inclusion of comprehensive and high-quality data. Additional references were identified through manual searches of bibliographies from selected papers. This systematic approach aimed to provide a thorough understanding of the multifaceted factors influencing extubation decisions in the context of pediatric cardiac surgery. Table 1 shows a list of included studies in the review.

**Table 1:** List of included studies in the review

Author(s)	Year	Focus
Rao SG [1]	2007	Overview of pediatric cardiac surgery in developing countries. This study provides a comprehensive review of the challenges, advancements, and outcomes of pediatric cardiac surgeries in developing nations, highlighting the disparities in healthcare access and resources.
Khalil M et al. [2]	2019	Acute therapy for newborns with critical congenital heart disease. This paper discusses immediate medical interventions and therapeutic strategies used to stabilize newborns diagnosed with severe congenital heart conditions.
Robinson A [3]	2002	Discusses early extubation after pediatric heart surgery. The focus here is on the practices, benefits, and outcomes of early removal of endotracheal tubes in children post-cardiac surgery to enhance recovery and reduce complications.
Özalp Ş et al. [4]	2023	Factors influencing fast-track or early extubation after pediatric cardiac surgery. This study identifies and analyzes various factors that contribute to the feasibility and success of early extubation protocols in pediatric cardiac patients.
Wu K et al. [5]	2020	Experience with early extubation after pediatric congenital heart surgery in China. The paper provides insights and data from clinical experiences in China, focusing on the outcomes and methodologies of early extubation in pediatric heart surgery.
Bronicki RA, Chang AC [6]	2011	Postoperative management strategies for pediatric cardiac surgical patients. This comprehensive review covers the various strategies employed to manage and support pediatric patients following heart surgery, aiming to optimize recovery and outcomes.
Rooney SR et al. [7]	2019	Variability in extubation failure rates across hospitals after pediatric cardiac surgery. This study investigates the differences in extubation success and failure rates among different hospitals, aiming to identify patterns and potential areas for improvement.
Rooney SR et al. [8]	2020	Impact of extubation location on outcomes following pediatric cardiac surgery. This research explores how the location where extubation is performed (e.g., operating room vs. ICU) influences the clinical outcomes and recovery of pediatric cardiac patients.
Simonato M et al. [9]	2023	Impact of preoperative pulmonary hemodynamics and cardiopulmonary bypass on lung function in children with congenital heart disease. The paper examines how preoperative lung conditions and the use of cardiopulmonary bypass during surgery affect postoperative pulmonary function in children.
Ödek Ç et al. [10]	2016	Predictors for early extubation after pediatric cardiac surgery. This study identifies key predictors and criteria that can help determine which pediatric patients are suitable candidates for early extubation post-surgery.
Baehner T et al. [11]	2022	Benefits and outcomes of on-table extubation following pediatric cardiac surgery. The focus here is on the practice of extubating patients on the operating table immediately after surgery and its impact on patient recovery and outcomes.
Brugmann University Hospital [12]	2022	Investigates the influence of intraoperative fluid balance on adverse events. This research looks into how the management of fluid balance during surgery affects the incidence of adverse events in pediatric cardiac patients.
Poletto E et al. [13]	2022	Review of ventilation weaning and extubation readiness in pediatric ICU. This paper reviews the protocols and practices for weaning pediatric patients off mechanical ventilation and assessing readiness for extubation in the intensive care unit.
Epstein R et al. [14]	2022	Trends and outcomes related to time to extubation in pediatric postoperative cardiac patients. The study analyzes recent trends in the timing of extubation and its effects on patient outcomes in the context of pediatric cardiac surgery.
Simpao AF et al. [15]	2023	Impact of anesthesia and sedation exposure on neurodevelopmental outcomes in infants undergoing congenital cardiac surgery. This research examines how exposure to anesthesia and sedatives during surgery influences the long-term neurodevelopmental outcomes of infants.
Wise-Faberowski L et al. [16]	2014	Examines the effects of anesthesia on the developing brain in the context of pediatric cardiac surgery. The study investigates the potential impacts of anesthetic agents on brain development in young children undergoing heart surgery.
Dennhardt N et al. [17]	2020	Early administration of clotting factors to prevent postoperative bleeding after complex pediatric cardiac surgery. This paper discusses the use of clotting factors administered early in the postoperative period to reduce the risk of bleeding complications.
Elassal AA et al. [18]	2021	Factors affecting re-exploration for bleeding after cardiac surgery. The focus is on identifying and analyzing factors that lead to the need for re-exploration surgeries due to postoperative bleeding in pediatric cardiac patients.
Kameny RJ et al. [19]	2016	Management strategies for pediatric pulmonary hypertension during the perioperative period. This study reviews strategies for managing pulmonary hypertension in children undergoing heart surgery, aiming to improve perioperative outcomes.
Tirotta CF et al. [20]	2020	Outcomes and experiences with immediate extubation in pediatric patients after congenital cardiac surgery. The paper provides data on the outcomes and practical experiences with extubating patients immediately following their cardiac procedures.
Gal DB et al. [21]	2022	Guidelines for managing postoperative pain in children undergoing cardiac surgery. This paper offers guidelines and recommendations for effectively managing pain in pediatric patients post-heart surgery to enhance recovery and comfort.
Iguidbashian JP et al. [22]	2020	Enhanced recovery and early extubation outcomes with single-dose intravenous methadone after pediatric cardiac surgery. The focus is on the use of methadone for pain management and its impact on recovery and early extubation outcomes.
Cruz-Suárez GA et al. [23]	2023	Postoperative analgesic outcomes using erector spine plane block in pediatric cardiac surgery with sternotomy. This study evaluates the effectiveness of using an erector spine plane block for pain relief in children undergoing sternotomy for heart surgery.
Biçer M et al. [24]	2023	Retrospective study on predictors of extubation in the operating room after pediatric cardiac surgery. This retrospective study analyzes factors that predict successful extubation in the operating room immediately after pediatric cardiac surgery.
Chan JL et al. [25]	2018	Implementation and outcomes of a multidisciplinary protocol to improve extubation times after cardiac surgery. This paper discusses the development and results of a multidisciplinary approach to reduce extubation times in pediatric cardiac patients.
Dani A et al. [26]	2022	Risk factors for prolonged intubation after pediatric heart transplantation. The study identifies risk factors that contribute to prolonged intubation periods in children following heart transplantation surgery.
Tanaka K et al. [27]	1986	Respiratory care strategies for pediatric patients needing prolonged intubation after cardiac surgery. This older study reviews respiratory care techniques for managing children who require extended intubation post-heart surgery.
Shinkawa T et al. [28]	2018	Incidence and predictors for reintubation after immediate extubation following pediatric cardiac surgery. The focus is on the incidence of reintubation and the factors that predict the need for reintubation after immediate extubation.
Alghamdi AA et al. [29]	2010	Systematic review and meta-analysis with evidence-based recommendations for early extubation after pediatric cardiac surgery. This comprehensive review and meta-analysis provide evidence-based guidelines and recommendations for early extubation practices in pediatric cardiac surgery.

Physiological factors

Sustaining hemodynamic stability, which ensures sufficient cardiac function and perfusion, is crucial for the successful extubation process. Postoperative maintenance of stable cardiac rhythms is essential to prevent hemodynamic instability, as any disruptions in these rhythms can impact the patient's capacity to tolerate the extubation procedure [7]. Pulmonary function plays a pivotal role in the decision-making process regarding extubation, particularly in children with CHD undergoing cardiac surgery. The alterations in pulmonary hemodynamics and lung function in this patient population may lead to abnormalities in respiratory mechanics and gas exchange. Individuals with increased pulmonary blood flow may experience postoperative lung damage, manifesting as reduced lung compliance and potential respiratory complications. The readiness for extubation and successful postoperative recovery hinge significantly on the meticulous monitoring of pulmonary function and the optimization of respiratory mechanics [9].

The age and size of a patient are crucial factors in the decision-making process for extubation. Younger and smaller patients may exhibit distinct physiological responses to cardiac surgery and mechanical ventilation, influencing their ability to tolerate extubation. Research identifies older age and increased body weight as predictors of early extubation success in pediatric cardiac surgery [10]. Children with compromised respiratory function may necessitate prolonged mechanical ventilation after surgery, which can affect the timing of extubation and overall recovery [11].

**Figure 1:**
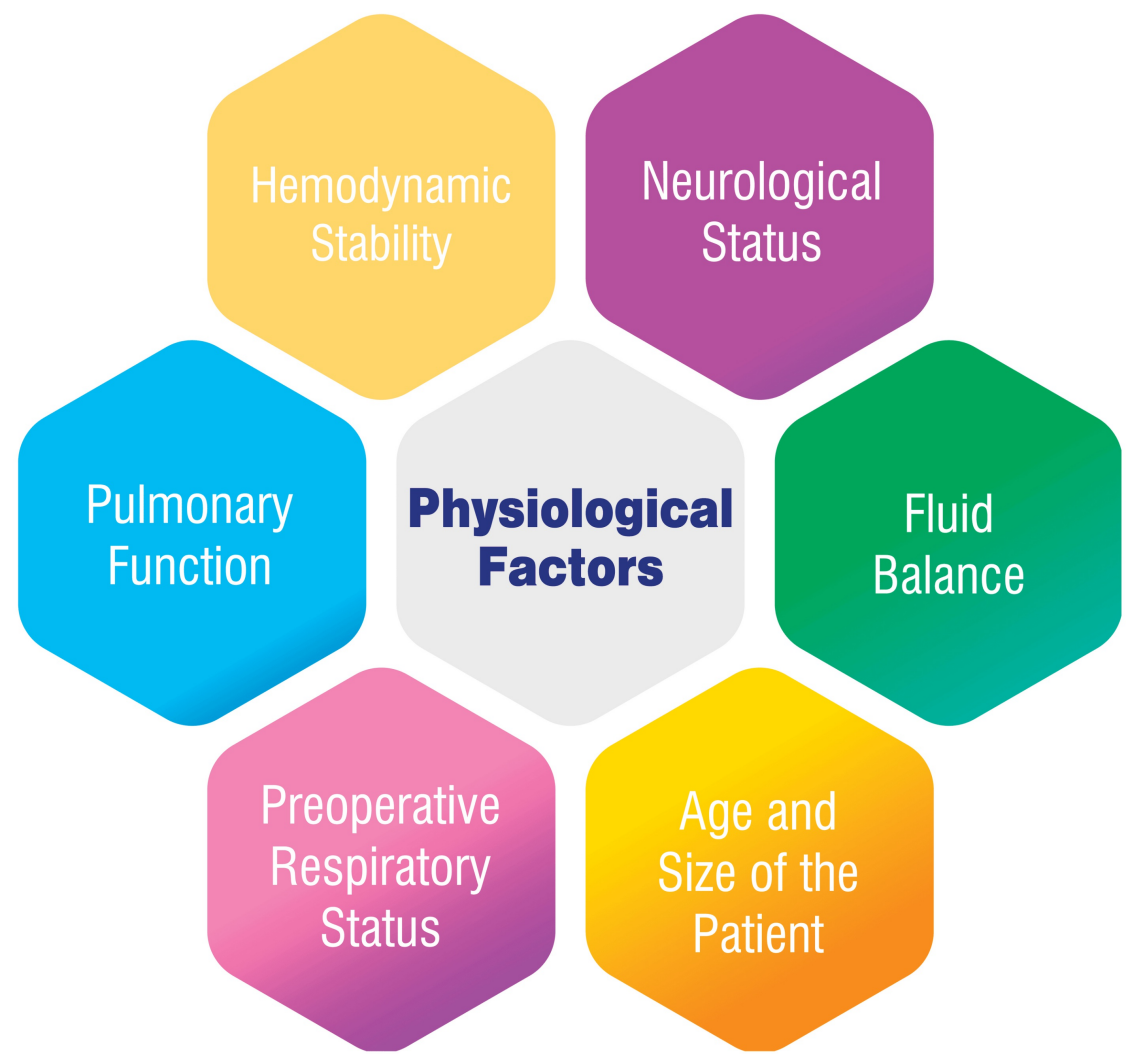
Physiological factors in extubation decision-making following pediatric cardiac surgery

Due to the unique challenges posed by the low circulation volume and complexity of pediatric cardiac surgery, maintaining an optimal intraoperative fluid balance is imperative. Precise control of fluid balance is crucial to prevent complications and enhance surgical outcomes, including the appropriate timing of extubation [12]. In children undergoing cardiac surgery, prolonged mechanical ventilation postoperatively can elevate morbidity and mortality rates while also adversely affecting neurodevelopment. Consequently, there is a trend toward promoting early extubation to mitigate these adverse consequences [10]. Assessing the neurological state is a critical aspect of determining extubation readiness. Patients with compromised neurological conditions may necessitate additional monitoring and care to facilitate successful extubation and ensure a smooth postoperative recovery [13]. Figure 1 shows physiological factors in extubation decision-making following pediatric cardiac surgery.

Surgical factors

The duration of mechanical ventilation and the success of extubation in pediatric patients may be subject to the complexity and duration of cardiac surgery. Prolonged mechanical ventilation and potentially elevated rates of extubation failure may be anticipated in the context of more complex surgical procedures. Patients characterized as early extubation, having undergone extubation either in the operating room or within six hours of admission to the pediatric ICU (PICU), exhibit associations with shorter ICU stays and diminished mortality rates. However, the relationship between surgical complexity and extubation outcomes is multifaceted, considering preoperative comorbidities, age, and weight [14].

In pediatric cardiac surgery, the residual presence of anesthesia constitutes a significant determinant in the decision-making process. Research conducted in some countries indicates a positive correlation between on-table extubation (OTE) during pediatric heart surgeries and favorable postoperative outcomes characterized by diminished durations of hospitalization and ICU stays [11]. However, children undergoing complex cardiac procedures may be susceptible to neurodevelopmental consequences arising from exposure to volatile anesthetic agents and sedative medications [15]. Despite ongoing investigations into the effects of anesthesia exposure on pediatric patients, it is paramount to consider the idiosyncratic attributes of each pediatric subject and carefully navigate a nuanced equilibrium between the advantages of early extubation and potential hazards associated with anesthesia exposure [16].

One crucial factor to consider when deciding on extubation protocols in pediatric heart surgery is postoperative bleeding. The definition of severe bleeding varies across medical centers due to differences in perioperative care methods, surgical complexity, and clinical expertise. Studies indicate that in children undergoing complex cardiac surgeries, early prophylactic administration of fibrinogen, prothrombin complex, and platelets-guided by clinical bleeding assessment and thromboelastographic-effectively enhances coagulation parameters and reduces bleeding occurrences [17,14]. The challenge lies in addressing severe bleeding, as considerable variability exists within medical centers, complicating establishing a universal definition. The research underscores the significance of prompt hemostatic interventions in effectively managing postoperative bleeding and minimizing the necessity for re-exploration due to bleeding, guided by a combination of clinical evaluation and thromboelastography [18].

**Figure 2:**
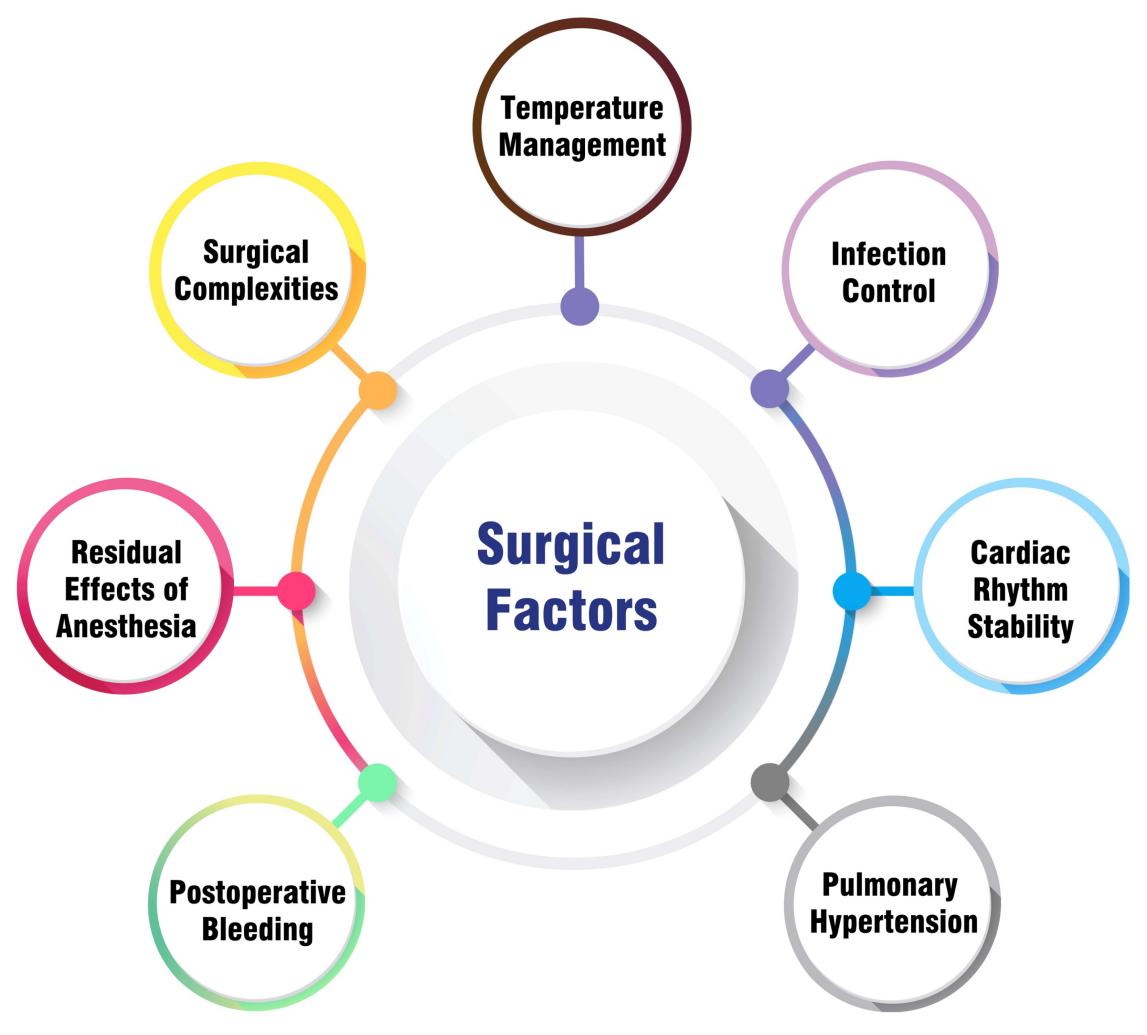
Surgical factors in extubation decision-making following pediatric cardiac surgery

Pulmonary hypertension, impacting hemodynamics and pulmonary vascular reactivity, poses challenges in perioperative management. Close monitoring is essential for patients with pulmonary hypertension throughout the perioperative period, especially during mechanical ventilation and the adaptation to altered hemodynamics post-surgery. The prevention of pulmonary hypertensive crises (PHCs) is critical due to their potential to induce sudden right ventricular failure and systemic hypotension. PHCs are marked by a sudden surge in pulmonary arterial pressure, leading to respiratory distress and unfavorable outcomes [19]. Temperature management represents another pivotal factor influencing extubation decisions in pediatric cardiac surgery. Research indicates that the safe accomplishment of early extubation within the initial 24 hours post-surgery is feasible in meticulously chosen patients, particularly those undergoing low- to medium-risk procedures [10]. Mitigating surgical complications, especially in pediatric patients with compromised immune systems, heavily relies on infection management. Implementing effective infection control measures is paramount to reduce the likelihood of infections that could prolong mechanical ventilation and delay extubation. This encompasses rigorous hand hygiene, adherence to aseptic procedures, and the judicious use of antibiotic prophylaxis [20]. Maintaining stable heart rhythms post-surgery is imperative to ensure adequate oxygenation and perfusion. Any irregular heart rhythm can lead to hemodynamic instability, compromising the patient's ventilation tolerance. To optimize extubation outcomes and prevent complications, continuous monitoring of cardiac rhythms is essential, and prompt intervention is necessary to manage arrhythmias [14]. Figure 2 shows surgical factors in extubation decision-making following pediatric cardiac surgery.

 

Postoperative care and support

Effective postoperative pain management significantly influences the decision to extubate pediatric patients following heart surgery. Despite the challenges and risks associated with opioids, they continue to be integral in appropriately controlling postoperative pain. Studies suggest that employing multimodal analgesic regimens, including methadone-based treatments, can facilitate early extubation with minimal side effects, leading to enhanced recovery for pediatric cardiac surgery patients [21,22]. Regional approaches, such as the ESPB, have been associated with reduced opioid usage and shorter ICU stays in pediatric patients undergoing heart surgery with sternotomy. These findings underscore the importance of implementing effective pain management strategies in expediting recovery and impacting the decision-making process for extubation after pediatric heart surgery [23].

**Figure 3:**
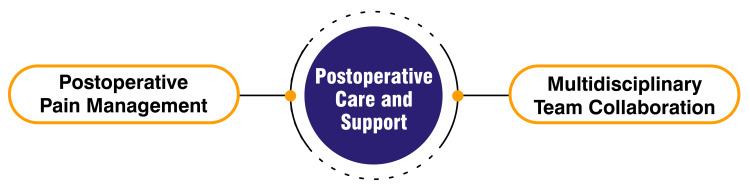
Postoperative care and support factors in extubation decision-making following pediatric cardiac surgery

A single-center retrospective analysis revealed that a consensus among the surgeons, cardiologists, and anesthesiologists was established before extubation in pediatric cardiac surgery patients [24]. Adopting a multidisciplinary, protocol-driven strategy has shown the potential to enhance extubation outcomes. This approach involves collaboration among healthcare experts to develop and adhere to a standardized extubation procedure. Implementing such a coordinated strategy has the potential to lead to improved patient outcomes and reduced complications in the context of pediatric cardiac surgery [25]. Figure 3 shows postoperative care and support factors in extubation decision-making following pediatric cardiac surgery.

Discussion

The decision to extubate pediatric patients following cardiac surgery is a crucial element of postoperative care. Christopher Tirotta et al., in their study, explained that immediate or early extubation strategies reduce morbidity, enhance functional status, and optimize the efficient utilization of healthcare resources. While mechanical ventilation was traditionally deemed essential for post-surgery patient stability, there is now a recognition that prolonged ventilation may elevate the risk of adverse outcomes during the postoperative period [20]. Studies, such as Robinson (2002), Wu et al. (2020), and Baehner et al. (2022), emphasize the benefits of early extubation protocols. These protocols are associated with reduced ICU stays, lower incidence of ventilator-associated complications, and overall improved recovery times [3,5,11]. The studies by Ödek et al. (2016) and Simonato et al. (2023) identify preoperative pulmonary hemodynamics and overall health status as critical factors influencing extubation readiness. Patients with better preoperative conditions are more likely to be extubated early [9,10].

The prolonged intubation in pediatric patients after cardiac surgery is strongly associated with multiple organ failure, underscoring its systemic impact. A well-established connection exists between extended endotracheal intubation and nosocomial infections, notably ventilator-associated pneumonia. These repercussions contribute to an extended hospital stay, potentially leading to long-term health consequences. Moreover, failure in the timely extubation process during pediatric cardiac surgery has been correlated with increased mortality rates, highlighting the critical importance of prompt extubation in mitigating these associated risks [26,27].

According to Takeshi Shinkawa et al., Enhanced Recovery After Surgery (ERAS) protocols have been successfully implemented across various medical fields, including adult cardiac surgery, to enhance clinical outcomes and decrease hospital stays. Although the adoption of ERAS concepts in pediatric cardiac surgery is not as widespread, research has investigated the effects of OTE following pediatric cardiac procedures. OTE has demonstrated promising results in reducing the duration of ICU treatment and overall length of stay in pediatric patients, suggesting its feasibility and potential benefits within this population [28]. However, while advantageous, immediate extubation in pediatric cardiac surgery poses potential risks, including reintubation, perioperative complications, increased risk in specific patient populations, and variability in outcomes, necessitating careful patient selection and evaluation [29].

Limitations

The complex interplay of variables in extubation decision-making following pediatric cardiac surgery presents several limitations. One significant challenge is the variability in patient-specific factors, such as age, underlying cardiac condition, and comorbidities, which can influence the extubation outcome. Additionally, differences in surgical techniques, postoperative care protocols, and institutional practices can result in inconsistent findings across studies. The subjective nature of clinical judgment in determining extubation readiness further complicates the standardization of criteria. Moreover, the lack of large-scale, randomized controlled trials limits the generalizability of existing evidence, necessitating more comprehensive research to establish universally applicable guidelines.

## Conclusions

In conclusion, the decision-making process for extubation following pediatric cardiac surgery is inherently complex and influenced by a myriad of variables. These include patient-specific factors such as age, underlying health conditions, and the type of cardiac surgery performed, as well as intraoperative and postoperative considerations like surgical complexity, hemodynamic stability, and the presence of complications. The benefits of early extubation, such as reduced length of stay, decreased ventilator-associated complications, and minimized exposure to sedation, underscore the importance of a nuanced and individualized approach. However, the successful implementation of early extubation protocols requires a thorough understanding of the intricate interplay of these variables. Ongoing research and advancements in perioperative care are essential to refine these protocols and improve outcomes for pediatric cardiac surgery patients.
